# Predictors of Overweight and Obesity and Its Consequences among Senoi Orang Asli (Indigenous People) Women in Perak, Malaysia

**DOI:** 10.3390/ijerph17072354

**Published:** 2020-03-31

**Authors:** Leh Shii Law, Norhasmah Sulaiman, Wan Ying Gan, Siti Nur’Asyura Adznam, Mohd Nasir Mohd Taib

**Affiliations:** 1 Faculty of Medicine and Health Sciences, Department of Community Medicine and Public Health, Universiti Malaysia Sarawak, Kota Samarahan 94300, Sarawak, Malaysia; lslaw@unimas.my; 2 Department of Nutrition and Dietetics, Faculty of Medicine and Health Sciences, Universiti Putra Malaysia, Serdang 43400, Selangor, Malaysia; wanying@upm.edu.my (W.Y.G.); asyura@upm.edu.my (S.N.A.); nasir.jpsk@gmail.com (M.N.M.T.)

**Keywords:** overweight, exercise, blood pressure, women, indigenous peoples

## Abstract

In spite of the high prevalence of overweight and obesity among the Orang Asli (OA) of Malaysia being an increasing concern due to the associated adverse health implications, information regarding this issue is scarce. This cross-sectional study is aimed to investigate the predictors of overweight and obesity and its association with blood pressure and quality of life among Senoi OA women. A total of 19 villages at Batang Padang, Perak, were selected out of a total of 56 villages using a simple random sampling, in which 355 Senoi OA women were participated in the study. Face-to-face interviews were conducted to obtain information on sociodemographic characteristics, dietary intake, physical activity, and quality of life. Weight, height and blood pressure were also measured. The prevalence of overweight and obesity were 32.4% and 26.2%, respectively. In terms of multiple linear regression, monthly household income, total energy intake, and metabolic equivalents (METs) for domestic activities were found to have significantly contributed to body mass index (BMI). Furthermore, BMI contributed significantly towards levels of blood pressure and quality of life after controlling for monthly household income, total energy intake, and METs for domestic activities. In conclusion, there should be urgent attention to poverty and overweight/obesity among the OA women. The findings would aid in alerting policy makers and health professionals as underweight is no longer a sole nutritional problem among OA but it appears to be coexisting with overweight and obesity. Strategies for improving their socioeconomic status, promoting a balanced and moderate diet, and encouraging involvement of OA women in physical activities should be implemented to prevent overweight and obesity.

## 1. Introduction

In Malaysia, indigenous peoples are given the specific name “Orang Asli” which in the Malay language means “First Peoples” or “Original Peoples” [1]. In accordance with the census data provided by the Department of Orang Asli Development (JAKOA) (unpublished), the total population of Malaysia’s Orang Asli (OA) in 2013 was 178,197, with the largest number of them residing in Pahang (67,506 people), followed by Perak (53,299 people) and Selangor (17,587 people). They are divided into three main ethnic groups: the Senoi, Proto-Malay and Negrito.

Although the OA represent a relatively small population, nutritional issues among them have been a growing concern. From a dietary aspect, previous studies have shown that OA communities have typically suffered from food insecurity [2,3]; micronutrient deficiencies such as lack of iron, iodine, protein, and vitamin A [4,5,6,7]; and unsatisfactory diet variety scores [8]. In addition, the OA were also found to have high incidence of overweight and obesity, abdominal obesity, reduced high-density lipoprotein cholesterol, and elevated triglyceride [8,9,10]. They were also found to be at higher risk of developing cardiovascular diseases and insulin-resistance to diabetes mellitus [11]. The focus of this study was on overweight/obesity due to its role as an important risk factor for non-communicable diseases [9] and due to the lack of information regarding the prevalence of this specific problem among the OA.

In Malaysia generally, the findings from the third National Health and Morbidity Survey (NHMS) in 2006 showed that prevalence of overweight and obesity among adults was 29.1% and 14.0%, respectively [12]. A slight increment was observed in the NHMS 2015, in which the prevalence of overweight and obesity were 30.0% and 17.7%, respectively [13]. On the other hand, prevalence of underweight among adults in the NHMS 2006 was at 8.5% [12] and this figure was reduced to 6.7% in 2015 [13]. Comparing these figures to independent studies conducted in both Pahang and Selangor in Malaysia among the OA, a double burden was revealed, where 26% of adult OA in Pahang were overweight and obese but as many as 49% and 64% of the children were underweight and stunted, respectively [10]. In addition, the study that was conducted in Selangor found that 31% and 20% of the OA women were overweight and obese, respectively, but 58% and 64% of the OA children were underweight and stunted [8].

High prevalence of overweight and obesity needs to be addressed urgently due to its devastating effects on physical and mental health. Overweight and obesity are closely associated with diabetes, high blood pressure, high cholesterol [14], cardiovascular diseases [15], and cancers [16]. In addition, being overweight and obese increases the risk of developing depression and anxiety [17]. Also, from an economic point of view, there are, of course, negative consequences of being overweight and obesity in terms of increased healthcare utilisation and escalated healthcare costs [18]. Furthermore, overweight and obese citizens tend to have lower workforce productivity [18].

Research on malnutrition among the OA has mostly been limited to demographic and socioeconomic status, involving factors such as ethnicity, sex of children, age of mothers, educational qualifications of mothers, household income per capita and number of children [10]. There has been little expansion to cover their dietary intake such as food variety scores for both women and children [8]. The lack of information regarding overweight and obesity among OA has restricted the adequate planning of intervention programs. Indeed, several highly relevant modifiable factors of overweight and obesity have not been studied at all among the OA, such as dietary behaviours (intake of sugar-sweetened beverages and heavy drinking) [19,20] and physical activity [21,22]. In addition, there are other modifiable predictors of overweight and obesity recognised as important in the literature, including psychological factors (body image perception and depression [23,24]) and built environment [25,26]. In order to fill in the knowledge gap regarding the overweight and obesity of Malaysia’s OA, therefore, this study not only involved demographic and sociodemographic characteristics but also covered physical activity and dietary factors among the OA.

This cross-sectional study aimed to investigate the relationships of demographic and socioeconomic characteristics, dietary intake and physical activity levels with body weight status among the Senoi OA women in Perak, Malaysia. In addition, this study also aimed to examine the relationship between overweight and obesity with blood pressure and quality of life. This exploratory study was planned in accordance with one of the objectives of the Plan of Action of Nutrition for Malaysia III to improve the nutritional well-being of Malaysians [27].

## 2. Literature Review

Previous studies investigating the nutritional issues of OA have demonstrated that OA adults are burdened with overweight and obesity. Mohd Adzim et al. [28] found that 28% and 23% of the OA adults living in Kuala Betis, Gua Musang, Kelantan, were overweight and obese, respectively. Chua et al. [29] revealed that the prevalence of overweight and obesity among OA adults at Krau Wildlife Reserve, Pahang, Malaysia was increased from 18.8% and 7.4% in 2011–2012 to 26.1% and 9.5% in 2015–2016. Furthermore, Nurfahilin and Normasmah [30] reported a high prevalence of overweight (30.4%) and obesity (29.3%) among OA women at Gombak, Selangor. Additionally, a local study conducted at the aborigine settlement in Sungai Ruil, Cameron Highlands, Pahang demonstrated 25.4% and 34.8% of overweight and obese OA adults, respectively [31].

Body weight status among OA has received increased attention as it is closely related to their health status. Overweight and obesity were found to be linked with hypertension [32] and diabetes mellitus [33] among OA adults. Overweight and obesity were linked to hypertension among the indigenous community at Dourados, Mato Grosso do Sul of Central Brazil [34]. Notably, non-communicable diseases such as hypertension, diabetes mellitus, insulin resistance and cardiovascular diseases [11,33] are linked to overweight and obesity in addition to the infectious diseases, including soil-transmitted helminth, dengue, Japanese encephalitis, and Zika [35,36]. Nevertheless, studies on the relationship between overweight and obesity with hypertension and quality of life in Malaysia have been scarce.

Demographic and socioeconomic characteristics that are found to be significantly associated with body weight status in females with younger age, more years of schooling, and reduced household income [29]. Another local study conducted by Wong et al. [10] also revealed that risk factors that included boy, older mothers, mothers with higher education, and households with income per capita less than USD 29.01 were significantly associated with a household with overweight/obese mother and stunted child. In the same study, being from the Jah Hut tribe was found to be protective against a household with overweight/obesity in mothers and an underweight and/or stunted child. The lower risk of double burden malnutrition is closely related to involvement of the Jah Hut tribe in economic activities such as rubber/oil palm cultivation and traditional food seeking behaviours such as farming, hunting, gathering, and fishing that have increased their physical activities level. A high risk of double-burden malnutrition among other tribes Che Wong and Temuan was due to close the distance of their resettlements to town, which has increased their access to processed food. Furthermore, Chong et al. [37] reported that food security was another leading factor for the occurrence of overweight and obesity among Mah Meri OA at Carey Island and Tanjung Sepat, Malaysia. Moreover, Nurul Hidayah et al. [38] reported that dietary acculturation was significantly linked with increased body mass index (BMI) among OA adults in Malaysia.

The overnutrition issue among OA warrants further investigation. A small-scale study could provide a holistic picture regarding the localized progression in the nutritional issues among OA. Literature review revealed several gaps: firstly, the research pertaining to the contributing factors towards nutritional status limited to sociodemographic and dietary factors; secondly, infectious diseases in addition to non-communicable diseases are prevalent among OA but the related studies have been scarce. Therefore, the current study was designed to fill in the gap by investigating the relationships between sociodemographic, dietary, and physical activity characteristics of the OA with overweight and obesity. Furthermore, the associations between overweight and obesity with blood pressure and quality of life were also examined.

## 3. Materials and Methods

### 3.1. Sampling

This was a cross-sectional study that involved only one ethnic group of OA, the Senoi. Based on the census data provided by JAKOA, the Senoi are the largest group among the OA of Malaysia when compared to the Proto-Malay and Negrito. The OA in Malaysia are not considered a homogenous group as a number of distinguishing factors are present pertaining to their language and culture [1,39]. It should be noted that most of the previous studies revealed a high prevalence of overweight and obesity among the Senoi OA. Hence, the Senoi group was selected as the target population in the current study in order to explore their nutritional issues. Perak was selected as the study location because Perak has the largest group of Senoi OA. Perak is the second largest state in Peninsular Malaysia and covers a landmass of 21,038 square kilometres [40]. Batang Padang, one of the 11 districts within the boundary of Perak, was randomly selected by using a random number generator (Microsoft Excel). During the selection process in the Batang Padang district, only 56 out of 80 villages that could be reached by a normal car were involved.

The average number of households for the 56 villages was 59.1. Based on the recruitment information from three villages, the mean probability to recruit eligible households was 31.4%. In order to achieve a calculated sample size at 336, the total number of households that needed to be approached was 1068.70. The number of villages needed was 18.1. Therefore, the number of villages required was 19. The 19 villages were selected randomly by using a random number generator (Microsoft Excel). The calculation is shown below:

Mean number of households in 56 villages: 3310/56

= 59.13 households

Eligible households from three villages: 31.4% of the total number of households

Total respondents that needed to be approached: (336 × 100)/31.44

= 1068.70 households

Number of villages needed to be selected: 1068.70/59.13

= 18.1 (approximately 19)

All of the eligible women from the selected villages were invited to participate in this study. Those OA women who were of childbearing age (20 to 49 years old) and having at least a child at home were included in this study. Pregnant or lactating women were excluded from this study.

### 3.2. Ethical Clearance and Permission

Ethical clearance was obtained from the Ethics Committee for Research Involving Human Subjects (JKEUPM) of the Universiti Putra Malaysia [Reference No: FPSK(EXP15)P004]. Permission to conduct research activities in the OA villages was granted by JAKOA. In addition, the respondents provided informed consent before data collection.

### 3.3. Data Collection

Data collection was conducted through face-to-face interviews, with the help of a questionnaire. The researcher was led by the *Tok Batin* (heads of the villages) or a highly-respected person in the selected villages and the research was conducted by travelling from house to house to meet the eligible respondents. Translators (local OA) were hired to help during interview process.

### 3.4. Study Instruments

#### 3.4.1. Demographic and Socioeconomic Characteristics

Information regarding the demographic and socioeconomic characteristics was collected via a questionnaire. Information included date of birth, date of data collection, villages, educational qualifications of the respondents and their spouses, monthly incomes of the respondents and their spouses, and total monthly household expenses. The household with a monthly household income of less than MYR 580 (USD 139.44) was categorised under hardcore poor status while poor status was used to describe a household with a monthly household income between MYR 580 (USD 139.44) to MYR 930 (USD 223.58) [41].

#### 3.4.2. Dietary Intake

Two days of 24-h dietary recall were conducted (one on a weekday and one on a weekend). The purpose of using this method was to gather information regarding all the food consumed in the preceding 24 h. The respondents were required to provide as much description as possible regarding all of the food items they had consumed, including the preparation methods for the food and the brand names of the processed food consumed. The amount of food consumed was measured using household measures in the form of standardised plastic cups and spoons. Later, the quantity of each food item consumed was converted into grams as a unit of measurement. The software used to analyse the total energy and nutrient intake was NutritionistPro 2.4.1 (First Data Bank Inc., South San Francisco, the United States). The food database reference used was mainly from the Nutrient Composition of Malaysian Food [42]. If the food was not available in the Nutrient Composition of Malaysian Food, other food database references such as the Canadian Nutrient File, the Nutrient Values of Alaskan Foods, and the United States Department of Agriculture (USDA) Standard Reference Database were used to obtain the nutrient values for the traditional foods of the OA such as frogs, squirrels and wild boars.

#### 3.4.3. Physical Activity

Physical activity levels for the previous seven days of the respondents were measured using the International Physical Activity Questionnaire (IPAQ) – long-form [43]. In total, the IPAQ has 27 items, but only 25 were used to determine activeness. These covered several domains, namely leisure-time physical activities, domestic and gardening (yard) activities, work-related physical activities, and transport-related physical activities. The respondents were required to report the number of days and duration of their involvement for each activity mentioned. The IPAQ protocol was referred to in order to calculate the metabolic equivalents (METs) for each domain [43]. A validated Malay-version of the IPAQ was used in this study [44].

#### 3.4.4. Quality of Life

The quality of life among respondents was assessed via the Medical Outcome Study Short Form 12 (SF-12) version 1 [45]. Eight physically and emotionally-based domains were measured, namely physical functioning, role limitations due to physical health, role limitations due to emotional problems, energy/fatigue, emotional well-being, social functioning, pain and general health. The raw scores provided information regarding mental and physical functioning. The Malay-version of the SF-12 was adapted from Malay-version SF-36 that was obtained from Ihab et al. [46]. In order to calculate the raw scores, the response on each item was converted into both physical and mental standardised values. To calculate physical component score (PCS), all of the physical standardised values from items 1 to 12 were summed. The summative scores were then added to 56.57706 to create PCS score [45]. Similarly, to calculate mental component score (MCS) scores, all of the mental standardised values were summed and the summative score was then added to a constant of 60.75781 to create an MCS score [45].

#### 3.4.5. Anthropometric Measurements

A TANITA Digital Weighing Scale HD-382 (TANITA Corporation, the United States) was used to measure weight to the nearest 0.1 kg. A SECA Bodymeter 206 (SECA, Germany) was used to measure height to the nearest 0.1 cm. Two measurements for weight and height were taken so that means could be calculated. With these means, the BMI for each respondent was then calculated using the classification of BMI from the World Health Organization (WHO) [47] to categorise the respondents according to their body weight status: BMI less than 18.5 kg/m^2^ = underweight; BMI between 18.5 to 24.9 kg/m^2^ = normal; BMI between 25.0 to 29.9 kg/m^2^ = overweight; BMI between 30.0 to 34.9 kg/m^2^ = obesity class I; BMI between 35.0 to 39.9 kg/m^2^ = obesity class II; BMI more than 40.0 kg/m^2^ = obesity class III.

#### 3.4.6. Blood Pressure

An electronic blood pressure meter HEM-7111 (Omron Healthcare, Japan) was used to measure the blood pressure of the respondents (systolic blood pressure [SBP] and diastolic blood pressure [DBP]). Blood pressure classification [12] is shown in Table 1.

### 3.5. Data Analysis

Data analysis was conducted by using IBM SPSS Statistics version 22.0 (IBM Corp., Armonk, NY, USA). Numeric variables were presented in means and standard deviations while categorical variables were shown in count and proportion. Simple linear regression analysis was performed to investigate the relationship between demographic and socioeconomic characteristics, dietary intake and physical activity with BMI. This step was to identify any potential variables that had a *p*-value of less than 0.25 for further analysis. Multiple linear regression (stepwise method) was then performed by entering into the model all of the variables that were found to have a *p*-value of less than 0.25 during simple linear regression. Lastly, the relationships between BMI with blood pressure and quality of life were examined by using multiple linear regression (enter method) after controlling for potential confounders such as monthly household income, energy intake and physical activity. The level of significance was set at *p* < 0.05.

## 4. Results

A total of 415 respondents were approached and, from these, 355 respondents agreed to participate. The response rate was 85.7%. The mean age of the respondents was 34.29 ± 8.61 years old. There was not much disparity in terms of years attending formal education between respondents (5.44 ± 4.69 years) and their spouses (5.57 ± 4.49 years). A high percentage of the respondents (37.7%) and their spouses (31.0%) did not have any formal education. About four-fifths of the respondents (79.4%) were housewives. Half of the respondents (49.9%) were considered as hardcore poor while another 19.7% of the respondents were poor.

Being underweight was found to occur among only a very small proportion of the respondents (3.7%). However, the prevalence of overweight and obesity was reported to be 32.4% and 26.2%, respectively. Moreover, 20.9% of the respondents were suffering from hypertension. Regarding quality of life, the means for PCS and MCS were 50.29 ± 7.93 and 52.56 ± 7.41, respectively.

In addition, the mean for total energy intake was 1935 ± 534 Kcal. Only half of the respondents who were 20 to 29 (57.0%) and 30 to 49 (45.6%) years old achieved the recommended dietary intake (RNI) of Malaysia. Moreover, the most common active physical activities that the respondents were involved in were domestic activities (1190.00 MET-minutes/week), followed by transportation (231 MET-minutes/week). Overall, about three quarters of the respondents were moderately active (71.3%). Further characteristics of the respondents are shown in Table 2.

The multiple linear regression showed that monthly household income (β = 0.115, *p* = 0.028), total energy intake (β = 0.131, *p* = 0.012) and METs for domestic activities (β = −0.335, *p* < 0.001) were found to be significant contributors to BMI. These three factors explained 14.6% of variance in BMI (Table 3).

Furthermore, BMI was found to have a significant contribution towards both blood pressure (SBP: β = 0.185, *p* = 0.001; DBP: β = 0.289, *p* < 0.001) and quality of life (PCS: β = −0.229. *p* < 0.001; MCS: β = −0.205, *p* < 0.001) after controlling for monthly household income, total energy intake and METs for domestic activities (Table 4).

## 5. Discussion

Overall, it was found that the standard of living among the OA women of this study was low due to their high poverty rate. The prevalence of overweight and obesity among the OA women were unexpectedly high. Factors that included their demographic and socioeconomic (monthly household income) status, their dietary intake (total energy intake) and their amount of physical activity (METs for domestic activities) were all found to make a significant contribution towards their BMI. On the other hand, BMI was also found to be a predictor for blood pressure (systolic and diastolic) and quality of life (PCS and MCS). Identification of these modifiable factors was vital for this study to improve understanding about the living conditions of OA.

In Malaysia, the OA are given a status as Bumiputera, together with the Malays and natives in Sabah and Sarawak. Under Bumiputera policy, the OA are endowed with special rights pertaining to economic, political, and social rights in education, representation in government, and political dominance (Nicholas, 2004). Nevertheless, the OA are still identified as a disadvantageous group under the 11th Malaysia Plan (2016–2020) [48]. A series of strategies has been implemented by the government to improve the conditions of life among the OA. Firstly, eradication of poverty was implemented under the Malaysia Plans, New Economy Policy, and Government Transformation Program [49] facilitated the decrease in the poverty rate of OA from 83.4% in year 2000 to 31.2% in 2010 (unpublished data from JAKOA). Free education is also provided to the OA children, including provision of free accommodation, school uniforms, and textbooks [50]. Despite the great achievement, the disparity in household income between the respondents of this study and the B40 group of Malaysia (those with a household income of MYR 2,537 [USD 606] in 2014) was still huge (the B40 group in Malaysia refers to households with an income in the bottom 40% of the population). Possible explanations for the high poverty rate of the OA were the remoteness of their settlements, being far from urban areas, poor educational qualifications, self-employment (mostly on small-scale agricultural production) and large household size [51].

Besides that, the efforts to tackle nutrition-related problems such as wasting and stunting, the community feeding program [52] and the program for the rehabilitation of malnourished children [53] under Ministry of Health Malaysia are carried out to improve the nutritional status of OA children, especially for those under 5 years of age. Nevertheless, the prevalence rates of underweight and stunting remain high [33], while a dual burden of malnutrition in OA households starts to emerge [10]. Similarly, Nurfaizah et al. [8] and Nurul Hidayah et al. [38] reported a high prevalence of overweight and obesity. Despite being a minority in Malaysia, the prevalence of overweight and obesity among the OA was as high as that of the general Malaysian population, where 30.0% overweight and 17.7% obesity were reported in the NHMS 2015 [13]. This provides evidence that the issues related to body weight status among the OA require urgent attention.

The most important predictor for overweight and obesity among OA women in this study were METs for domestic activities such as gardening, cleaning the yard and washing clothes by hand. High METs for domestic activities appeared to be protective against overweight and obesity. The inverse relationship between domestic activities and body weight status was supported by Banks et al. [54], where the risk of obesity was reduced in line with the increase in the frequency of household chores and gardening. However, Murphy et al. [55] presented contradictory findings, stating that time spent on domestic activities was negatively associated with leanness. Indeed, the negative relationship between METS for domestic activities and BMI depends very much on the nature of the domestic activities undertaken. Unlike most of the general population, the OA tend to carry out domestic activities manually without relying on labour-saving devices such as washing machines or vacuum cleaners [54].

Monthly household income showed a positive relationship with BMI in this study. This finding was not supported by most of the previous studies [56,57], in which they revealed that household income was inversely associated with BMI among women. One possible explanation for these contradictory findings is the difference in the respondents; the OA of the present study often having marked differences in culture, beliefs and values. Indeed, the household income could have an impact on overweight and obesity among the OA due to nutrition transition (changing patterns in food consumption) caused by personal wealth; a shift from consuming traditional food to a high consumption of refined grain, meat, and edible oil [58,59]. It can be concluded, therefore, that socioeconomic factors have influenced the food choices of the OA and that the resulting unhealthy selection of food has led to abnormal body weight status.

Another important predictor of overweight and obesity among OA women in this study was total energy intake. Total energy intake was found to contribute positively to BMI, which is consistent with the findings of Shankar [60] and Moon et al. [61]. The positive findings of this study could be due to the presence of a large number of OA from mild and moderate household food insecurity groups, which could be predicted because of a high rate of poverty. The relationship between household food insecurity and overweight and obesity is complex, as only severe household food insecurity was found to contribute to underweight while mild to moderate household food insecurity, on the other hand, led to overweight and obesity [62]. However, no causal conclusion could be drawn from this finding because there was no evidence indicating whether high energy intake is linked with high BMI or high BMI requires more energy to maintain the needs of extra mass and for preventing weight loss [61].

Regarding the consequences of overweight and obesity, the positive relationship between BMI and blood pressure (both SBP and DBP) is supported by previous studies [63,64]. Wang et al. [63], for example, emphasised that overweight adults possessed a higher risk of developing hypertension, while Julius et al. [64] revealed that overweight and weight gain were associated with the occurrence of hypertension. The interaction between BMI and hypertension is believed to be explained by the sympathoadrenal system that prioritises the primacy of eating behaviours in the determination of obesity, insulin resistance, and metabolic abnormalities that later turn into the occurrence of hypertension [65].

Moreover, BMI in this study was found to have a negative association with quality of life (both PCS and MCS). This finding is supported by Ghorbani et al. [66], where scores for six domains for SF-36 were found to be significantly different between three groups of BMI. Similarly, Giuli et al. [67] reported that BMI was negatively associated with physical subscales of SF-36. The negative correlation between BMI and quality of life is due to the presence of pathologies (type II diabetes, high blood pressure, and non-alcoholic fatty liver) that impair the quality of life among the respondents who were overweight and obese [67].

Several limitations were found in this study. The first being that it applied a cross-sectional study design, meaning that causal inferences could not be concluded since all the measurements were taken at a single point of time and the temporal relationships from the cross-sectional study were not consistent. Secondly, the scope of this study was limited to only involve one particular group of OA, the Senoi OA women from regions with connections to nearby towns by roads. Hence, the findings of this study cannot be generalised to other OA living in different environments. As such, it is recommended that future studies should be designed to involve respondents living in remote areas and from all the three ethnic groups of OA: the Senoi, Proto-Malay and Negrito. Also, future studies could involve both male and female OA so that comparisons between male and female OA could be drawn. It should also be remarked that dependency on a self-reported questionnaire such as SF-12 and IPAQ is prone to the occurrence of response bias (socially desirable responding, acquiescent responding, and extreme responding) [68].

## 6. Conclusions

Notably, OA are facing social and health issues such as a high poverty rate and also a high prevalence of overweight and obesity in their communities. This suggests that the development programs for OA need to be continued in order to further improve their disadvantageous conditions. The findings of this study also revealed the presence of strong predictors (monthly household income, total energy intake, and METs for domestic activities) and consequences (blood pressure and quality of life) of the occurrence of overweight and obesity among the OA. The discovery of such predictors of overweight and obesity in these communities could be used as a reference in the planning of health education programs in order to increase the effectiveness of such programmes. There certainly needs to be a strong focus during health promotion and intervention programs on ways of improving socioeconomic characteristics, promoting a well-balanced and moderate diet, and encouraging involvement in more physical activities. The important consequences of overweight and obesity also mean that special care is needed to ensure that those with these problems and, especially, hypertension receive the necessary treatment so that their daily routines are not adversely affected.

## Figures and Tables

**Table 1:** Classification of blood pressure for adults aged 18 years and older.

Category	Systolic Blood Pressure (mmHg)	Diastolic Blood Pressure (mmHg)
**Optimum**	<120 and	<80
**Normal**	<130 and	<85
**High normal**	130–139 and/or	85–89
**Hypertension**		
**Stage I**	140–159 and/or	90–99
**Stage II**	160–179 and/or	100–109
**Stage III**	≥180 and/or	≥110

Source: Institute of Public Health, IPH [12].

**Table 2:** Characteristics of the respondents (*n* = 355).

Variable	n	%	Mean ± SD
Demographic Characteristics			34.29 ± 8.61
Age (years)			
20s	114	32.1	
30s	136	38.3	
40s	105	29.6	
Religion			
Islam	22	6.2	
Christianity	148	41.7	
Animism	124	34.9	
Bahai	61	17.2	
Marital status			
Married	322	90.7	
Widow	33	9.3	
Household size			
≤3	73	20.6	
4–7	231	65.1	
≥8	51	14.4	
Number of children			
≤3	261	73.5	
4–7	81	22.8	
≥8	13	3.7	
Socio-Economic Status			
Education qualification of respondents (year)			5.44 ± 4.69
No formal education	134	37.7	
Primary school	66	18.6	
Secondary school	150	42.3	
Pre-U	3	0.8	
University	2	0.6	
Education qualification of spouses (year)			5.55 ± 4.48
No formal education	108	30.4	
Primary school	73	20.6	
Secondary school	135	38.0	
Pre-U	3	0.8	
University	3	0.8	
Passed away/divorced	33	9.3	
Occupation of respondents			
Professionals	2	0.6	
Technician and associate professionals	2	0.6	
Clerical support workers	1	0.3	
Service and sale workers	8	2.3	
Skilled Agricultural, Forestry, and Fishery Workers	38	10.7	
Plant and Machine-operators and Assemblers	5	1.4	
Elementary occupations	17	4.8	
Others			
Housewife	282	79.4	
Occupation of spouses			
Managers	7	2.0	
Professionals	1	0.3	
Technician and associate professionals	11	3.1	
Clerical support workers	1	0.3	
Service and sale workers	27	7.6	
Skilled agricultural, forestry, and fishery workers	166	46.8	
Craft and related trades workers	19	5.4	
Plant and machine operators assemblers	19	5.4	
Elementary occupations	55	15.5	
Armed forces	7	2.0	
Others			
Retired	3	0.8	
Passed away	16	4.5	
Divorced	17	4.8	
No working	6	1.7	
Monthly household income (MYR) *			817.28 ± 753.93
Hardcore poor (≤MYR580)	177	49.9	
Poor (>MYR580 and ≤MYR930)	70	19.7	
Normal (>MYR930)	108	30.4	
Household income per capita (MYR) *			176.71 ± 180.27
Hardcore poor (≤MYR140)	210	59.2	
Poor (>MYR140 and ≤MYR230)	57	16.1	
Normal (>MYR230)	88	24.8	
Expense on necessities (MYR)			385.41 ± 211.93
≤299.99	107	30.1	
300.00–599.99	196	55.2	
≥600.00	52	14.6	
Dietary Intakes			
Calorie (Kcal)			1935 ± 534
20 to 29 years old			1988 ± 529
< recommended dietary intake (RNI) (1840 Kcal)	49	43.0	
≥ RNI	65	57.0	
30 to 49 years old			1910 ± 535
<RNI (1900 Kcal)	131	54.4	
≥RNI	110	45.6	
Carbohydrate (g/d)			278.80 ± 74.60
<50%	51	14.4	
50%–65%	238	67.0	
>65%	66	18.6	
Protein (g/d)			80.74 ± 30.44
<10%	11	3.1	
10%–20%	291	82.0	
>20%	53	14.9	
Fat (g/d)			54.53 ± 22.16
<25%	177	49.9	
25%–30%	115	32.4	
>30%	63	17.7	
Physical Activity			
Domain Sub Scores (Metabolic Equivalents (MET)-minutes/week)			
Work			0
Transportation			99.00
Domestic and garden			1070.00
Leisure-time			0
Total Physical Activity Score (MET-minutes/week)			1728.00
Physical Activity Level			
Low	14	3.9	
Moderate	253	71.3	
High	88	24.8	
Anthropometric Measurements			
Body mass index (kg/m^2^)			26.53 ±5.38
Underweight	13	3.7	
Normal	134	37.7	
Overweight	115	32.4	
Obesity	93	26.2	
Blood pressure (mmHg)			
Mean systolic pressure			126.18 ± 15.91
Mean diastolic pressure			78.74 ± 11.53
Optimum	121 (34.1)		
Normal	87 (24.5)		
High normal	73 (20.6)		
Hypertension			
Stage I	57 (16.1)		
Stage II	12 (3.4)		
Stage III	5 (1.4)		
Quality of Life			
Physical component score (PCS)			50.31 ± 7.92
Mental component score (MCS)			52.49 ± 7.45

* Classification of poverty status was based on the guidelines by Economic Planning Unit (2014).

**Table 3:** Correlations and contributions of demographic and socioeconomic status, dietary intake, and physical activity with body mass index (BMI, *n* = 355).

Variable	Simple Linear Regression			Multiple Linear Regression a		
	β/rs	t	*p*	β	t	*p*
Demographic and socioeconomic status						
Age	0.053	0.999	0.319			
Number of children	0.038	0.719	0.472			
Household size	−0.009	−0.173	0.863			
Year of education (respondents)	0.054	1.021	0.308			
Year of education (spouses)	0.072	1.301	0.194			
Monthly household income	0.152	2.882	0.004	0.115	2.204	0.028
Monthly income per capita	0.126	2.387	0.017			
Total monthly expense	0.137	2.599	0.010			
Dietary Intake						
Total Energy intake	0.158	3.013	0.003	0.131	2.514	0.012
Carbohydrate intake	0.142	2.698	0.007			
Protein intake	0.150	2.850	0.005			
Fat intake	0.126	2.393	0.017			
Physical Activity						
METs for work b	−0.043		0.422			
METs for transportation b	−0.188		<0.001			
METs for domestic activities b	−0.308		<0.001	−0.335	−6.471	<0.001
METs for leisure activities b	0.019		0.715			

a Adjusted R^2^ = 0.146; F = 18.110; *p* < 0.001; b Spearman’s rank order correlation.

**Table 4:** Contributions of BMI towards blood pressure and quality of life.

Variable	Simple Linear Regression			Multiple Linear Regression		
	β	t	*p*	β	t	*p*
Systolic Blood Pressure						
BMI *	0.224	4.317	<0.001	0.185	3.312	0.001
Diastolic Blood Pressure						
BMI *	0.282	5.527	<0.001	0.259	4.728	<0.001
Physical Component Score						
BMI *	−0.239	−4.616	<0.001	−0.229	−4.129	<0.001
Mental Component Score						
BMI *	−0.188	−3.605	<0.001	−0.205	−3.663	<0.001

* after controlling for monthly household income, total energy intake, and METs for domestic activities.
